# Systemic treatment strategies for BRAF wild-type metastatic unresectable melanoma: a network meta-analysis

**DOI:** 10.3389/fonc.2026.1967814

**Published:** 2026-10-02

**Authors:** Yishan Zhou, Yuanmin He, Xiaoqi Tan

**Affiliations:** Skin Structure and Function Key Laboratory of Luzhou, Department of Dermatology, The Affiliated Hospital, Southwest Medical University, Luzhou, Sichuan, China

**Keywords:** BRAF wild-type, melanoma, metastatic unresectable, network meta-analysis, systemic treatment

## Abstract

**Background:**

This study aimed to provide new insights into systemic treatment for BRAF wild-type metastatic unresectable melanoma, thereby offering a basis for personalized clinical decision-making.

**Methods:**

PubMed, Embase, Cochrane Library, and Web of Science were searched. Eligible participants were adults with pathologically confirmed BRAF V600 wild-type metastatic unresectable melanoma. Outcomes included progression-free survival (PFS) and overall survival (OS). Network meta-analysis (NMA) results were presented as league tables and the surface under the cumulative ranking curve (SUCRA) values.

**Results:**

In total, 8 randomized controlled trials (RCTs) involving 3,418 patients and 9 systemic treatment strategies were included. One open-label study was rated as having some concerns, while the remaining seven were rated as low risk of bias across all domains and overall. The NMA results showed that, compared to ipilimumab, pembrolizumab, nivolumab, nivolumab + relatlimab, and nivolumab + ipilimumab demonstrated significant advantages in improving OS. Notably, nivolumab + relatlimab ranked first in prolonging OS (SUCRA = 93.94%). In the PFS analysis, this regimen had the highest SUCRA value only within the connected network consisting of dacarbazine, nivolumab, and the combination of nivolumab and relatlimab (SUCRA = 98.86%), although no comparisons or standardized rankings were performed between different PFS networks.

**Conclusions:**

The results of OS primary analysis for nivolumab plus relatlimab, as well as its ranking within the corresponding PFS network, are exploratory in nature. They are intended solely to generate research hypotheses and should not be interpreted as evidence of superiority over other treatments or as a basis for changing the current standard of care. Direct head-to-head RCTs are still needed to further clarify the comparative efficacy of the relevant regimens.

## 1 Background

Melanoma represents a highly aggressive malignancy arising from cutaneous melanocytes. Though it accounts for less than 5% of all malignancies worldwide, melanoma has persistently ranked among the foremost contributors to skin cancer-associated mortality owing to its rapid progression, strong propensity for metastasis, and poor responsiveness to conventional therapies (1, 2). Based on data from the Global Cancer Observatory 2021, approximately 320,000 new cases of cutaneous malignant melanoma and over 50,000 deaths are reported annually worldwide. Moreover, owing to increasing ultraviolet exposure and population aging, the incidence of melanoma in certain regions continues to rise. This trend imposes a substantial public health burden (3, 4).

Though the prognosis of patients with localized melanoma has improved markedly due to advances in early screening and local therapeutic strategies, clinical outcomes for metastatic unresectable melanoma remain suboptimal. Prior to the advent of immunotherapy, the median overall survival (OS) for these populations was typically solely 6–9 months, with a 5-year survival rate of less than 10%. This unfavorable prognosis severely compromises patients’ quality of life and imposes a substantial burden on healthcare systems (5, 6). Therefore, metastatic unresectable melanoma ranks among the most challenging clinical subtypes in the therapeutic field of melanoma.

With the advances in molecular subtyping and tumor immunology, systemic therapy for melanoma has increasingly adopted precision medicine approaches. For individuals harboring BRAF V600 mutations, targeted therapy combining BRAF and MEK inhibitors can significantly improve tumor control rates and survival outcomes. Furthermore, it might serve as the standard first-line treatment for this population (7, 8). However, for individuals with BRAF wild-type metastatic unresectable melanoma, clinical management has traditionally relied on non-specific therapies due to the absence of targetable driver mutations. Consequently, clinical options remain limited, and the optimal systemic treatment strategies remains controversial (7, 8).

Recently, the emergence of immune checkpoint inhibitors (ICIs) has profoundly revolutionized the clinical management of BRAF wild-type metastatic unresectable melanoma. Immunotherapy monotherapy, represented by PD-1 inhibitors such as nivolumab and pembrolizumab, has shown notable efficacy in improving progression-free survival (PFS) and OS compared to conventional chemotherapy. Consequently, these agents have gradually replaced dacarbazine as a relevant systemic treatment strategy. Based on this situation, numerous immunotherapy-based combination strategies have emerged, including PD-1 inhibitors combined with CTLA-4 inhibitors, PD-1 inhibitors combined with novel ICIs (e.g., LAG-3), and regimens combining immunotherapy with antiangiogenic agents. This diversification significantly expands systemic treatment strategies for individuals with BRAF wild-type metastatic unresectable melanoma (9, 10).

Nevertheless, diverse immunotherapeutic regimens exhibit marked heterogeneity in clinical benefit, toxicity profiles, and tolerability of patients. For instance, though dual ICI combinations have demonstrated superior tumor control in certain studies, they are associated with a markedly elevated incidence of grade ≥ 3 immune-related adverse events. This condition limits their applicability in populations with poor performance status or underlying comorbidities (11). By contrast, ICI monotherapy offers a more favorable safety profile. The comparative long-term survival benefit of ICI monotherapy versus combination regimens remains inconclusive. Furthermore, though novel combination strategies (e.g., PD-1 inhibitors combined with LAG-3 inhibitors or anti-angiogenic agents) have shown potential advantages, the relative efficacy of these therapies in the systemic setting needs to be further investigated.

Currently, head-to-head comparisons regarding systemic treatment strategies for BRAF wild-type metastatic unresectable melanoma remain lacking. It is challenging for clinicians to systematically evaluate multiple treatment strategies. Traditional pairwise meta-analyses can solely synthesize direct evidence, failing to adequately evaluate the relative effects of multiple interventions. In contrast, network meta-analysis (NMA) simultaneously integrates both direct and indirect evidence, facilitating a comprehensive comparison and ranking of efficacy across multiple regimens. This approach provides more robust and comprehensive evidence to support complex clinical decision-making.

This research employs an NMA to systematically synthesize previously published randomized controlled trials (RCTs). Focusing on systemic treatment strategies for individuals with BRAF wild-type metastatic unresectable melanoma, we aimed to compare the relative efficacy of immunotherapy monotherapy, dual-immunotherapy combinations, and immunotherapy-based combination strategies regarding PFS and OS. By systematically assessing the efficacy profiles of diverse regimens, this research sought to provide a clearer and more reliable evidence-based foundation for systemic treatment strategies, ultimately informing individualized clinical decision-making.

## 2 Methods

This research followed the Preferred Reporting Items for Systematic Reviews and Meta-Analyses (PRISMA) statement and the PRISMA extension for NMA (12, 13). The protocol was pre-registered on PROSPERO (registration number: CRD420251186147).

### 2.1 Inclusion and exclusion criteria

Inclusion criteria were as follows: (i) participants (P) were individuals aged ≥ 18 years with pathologically confirmed BRAF V600 wild-type metastatic unresectable melanoma; (ii) interventions (I) were systemic treatment strategies, including PD-1/PD-L1 inhibitor monotherapy, combinations of ICIs, immunotherapy + chemotherapy, or immunotherapy + anti-angiogenic agents; (iii) comparators (C) involved conventional regimens (e.g., dacarbazine monotherapy or other standard therapies); (iv) outcomes (O) included at least PFS, OS; (v) study design (S) was RCTs published in English. This study included RCTs of systemic therapy for BRAF wild-type metastatic unresectable melanoma. Some studies allowed for a limited history of systemic therapy, not strictly limiting enrollment to patients who had received no prior systemic therapy.

Exclusion criteria were as follows:

(i) systematic reviews, meta-analyses, commentaries, and guidelines; (ii) conference abstracts and study protocols; (iii) animal experiments, *in vitro* studies, and mechanistic studies; (iv) non-RCT designs (encompassing cohort studies and case reports); (v) studies including individuals with BRAF mutations where data for the BRAF wild-type subgroup could not be extracted separately; (vi) studies with incomplete data or those from which survival curves and effect sizes could not be extracted; key information could not be obtained by contacting the authors; (vii) duplicate publications.

### 2.2 Literature search and study selection

PubMed, Embase, Cochrane Library, and Web of Science were searched from database inception to September 4, 2026. The search strategy was designed employing a combination of subject terms and free terms related to ‘Neoplasm Metastasis’, ‘Melanoma’ and ‘Randomized Controlled Trial’. These terms were then combined using Boolean operators (AND/OR) and tailored to the specific requirements of each database. The primary search strategy was ‘Neoplasm Metastasis’ AND ‘Melanoma’ AND ‘Randomized Controlled Trial’. Detailed search strategies are provided in **Supplementary Table 1**.

The process of study selection was conducted independently by two researchers. All retrieved records were imported into EndNote 2025 for deduplication to ensure that each study appeared only once in EndNote. Initial screening was conducted by reviewing titles, abstracts, and keywords based on pre-specified inclusion and exclusion criteria. Subsequently, the full texts of potentially eligible studies were retrieved and reviewed for final inclusion. Any divergences were resolved through discussions and re-examination of the original articles, under the guidance of a third researcher.

### 2.3 Data extraction

Data extraction was categorized into three primary components: (i) general study characteristics, including first author, publication year, country, sample size, age, sex, and the severity of diseases, disease stage, Eastern Cooperative Oncology Group (ECOG) performance status, lactate dehydrogenase (LDH) levels, brain metastases, PD-L1 expression; (ii) details of interventions and controls, encompassing name of treatment, sample size, dosage, route of administration, frequency, and duration of therapy; (iii) outcome measures, including hazard ratios (HRs) and 95% confidence intervals (CIs) for PFS and OS, and the objective response rate (ORR). This study focused primarily on the analysis of efficacy. Data were extracted independently by two researchers. Any divergences were resolved through consultation with a third researcher.

### 2.4 Quality assessment

The methodological quality of all included studies was assessed independently by two reviewers using the Risk of Bias (RoB) 2.0 tool for RCTs. As a well-established tool for assessing RCTs, it evaluated risk of bias across five domains: bias arising from the randomization process, bias due to deviations from intended interventions, bias owing to missing outcome data, bias in measurement of the outcome, and bias in selection of the reported result (14). For each theme, the risk of bias was categorized as low risk of bias, some concerns, or high risk of bias. The overall risk of bias for each study was also assigned to one of these three categories. Any divergences between the two reviewers during the scoring process were resolved through arbitration by a third researcher to ensure the objectivity and consistency of the assessment.

### 2.5 Data analysis

This study used the gemtc package in R (version 4.5.2) to invoke JAGS for conducting a Bayesian fixed-effects NMA assuming consistency. The fixed-effects model assumes that the true effect for each treatment comparison is identical across studies and does not estimate between-study heterogeneity. The basic treatment effect parameter was assigned a zero-mean normal prior with a standard deviation of 15 times the model scale parameter (om.scale), which was determined by the software based on the data. Four Markov chains were run, each with 5,000 burn-in iterations followed by 20,000 sampling iterations (thinning interval = 1). Gelman–Rubin diagnostics and trajectory plots were used to assess convergence. The potential scale reduction factor (PSRF) for all monitored parameters was 1.00, indicating adequate convergence. Comparisons between treatments were presented in a network diagram. To assess the sensitivity of OS results to model specifications, a separate random-effects model was fitted to estimate the between-study standard deviation τ, using a Uniform (0, om.scale) prior. The posterior median of τ and its 95% credible intervals (CrIs) were reported, and the effect estimates and DIC values for the fixed-effects and random-effects models were compared. For PFS, fixed-effects models were used. For OS, after excluding a study with a follow-up duration of 10 years, fixed-effects models were fitted separately to each connected component of the OS network. As no closed loops were formed in the network, the node-splitting method for inconsistency testing was not performed. The primary results were presented using league tables and surface under the cumulative ranking curve (SUCRA) values. The league tables presented HRs with 95% CrIs for the relative survival benefits of different interventions. An HR < 1 indicated a more effective regimen. Given the absence of closed loops and the presence of a disconnected network structure, the SUCRA rankings in this study were used primarily for the generation of hypothesis rather than direct clinical ranking. A SUCRA value closer to 100% indicated a higher probability of better therapeutic efficacy. The network for the primary OS analysis was connected, whereas the sensitivity analysis for OS and the PFS analysis (excluding Wolchok 2025) contained disconnected components. SUCRA rankings are interpretable only within each outcome and each connected component. They are intended for exploratory description and hypothesis generation, and should not be used alone as a basis for clinical recommendations or for comparing treatments across disconnected components. The ORR analysis employed the same fixed-effects model framework, prior settings, and MCMC parameters as the OS and PFS analyses, with estimates based on the number of objective responses and the total number of patients in each study arm.

## 3 Results

### 3.1 Study selection

In total, 20538 studies were retrieved, specifically from PubMed (2258), Embase (12299), Cochrane Library (3526), and Web of Science (2455). Following the removal of 5,021 duplicates, the titles and abstracts of the remaining records were screened. Subsequently, 15462 studies were excluded. The full texts of the remaining 55 articles were assessed. Among them, 8 conference abstracts and 5 **Supplementary Materials** were excluded due to incomplete information. Excluded studies included 19 lacking BRAF wild-type results, 12 duplicates, 1 cohort study, 1 with inconsistent disease, and 1 with inconsistent intervention. Finally, 8 RCTs were included (15–22) (**Figure 1**).

**Figure 1:**
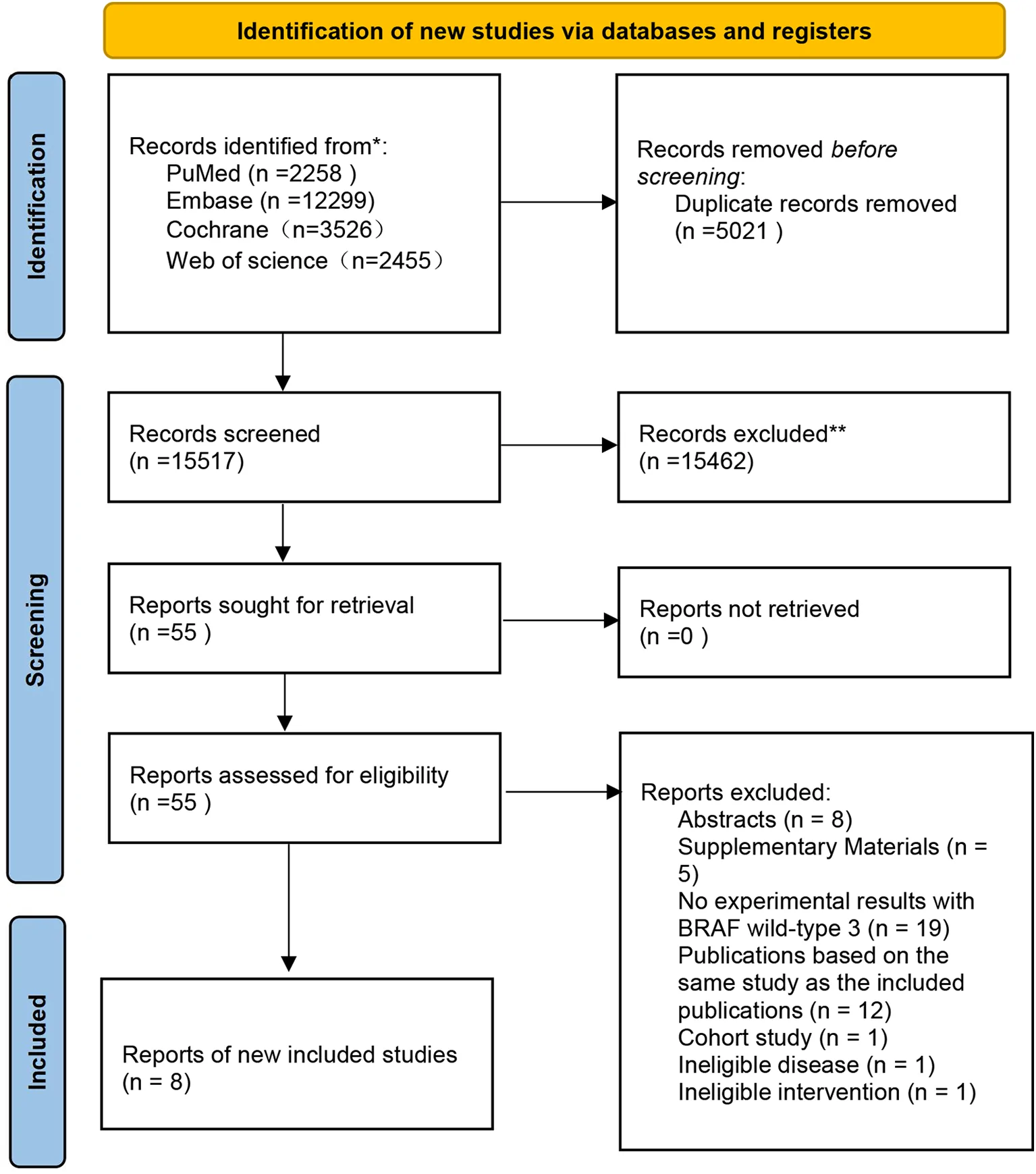
PRISMA flow diagram of literature screening.

### 3.2 Basic characteristics of included studies

The eight included studies were published between 2015 and 2025, with sample sizes ranging from 109 to 647. The total sample size was 3,418 participants. All were multicenter studies conducted in multiple countries. The median age ranged from 61 to 69 years. All patients had BRAF wild-type metastatic unresectable melanoma, and most had an ECOG PS score of 0–1. Nine systemic treatment strategies were involved: ipilimumab (4 studies), nivolumab plus ipilimumab (2 studies), pembrolizumab (3 studies), dacarbazine (1 study), nivolumab (3 studies), epacadostat plus pembrolizumab (1 study), ipilimumab 10 mg/kg (1 study), nivolumab plus relatlimab (1 study), and lenvatinib plus pembrolizumab (1 study). Detailed characteristics of included studies are provided in **Supplementary Table 2**. The core clinical characteristics at baseline were generally balanced across the studies, ensuring comparability of the study populations for conducting an NMA.

### 3.3 Risk of bias assessment

All 8 included studies were multicenter RCTs, which provided a robust foundation for efficacy comparisons. Based on an assessment using the RoB 2 tool, 7 studies were judged to have a low risk of bias in all domains and in the overall assessment. The remaining study was an open-label trial. Because information on treatment assignment could influence outcome assessment, both the outcome measurement domain and the overall risk of bias were judged to have some concerns. Overall, the risk of bias in the included studies was low (**Figure 2**).

**Figure 2:**
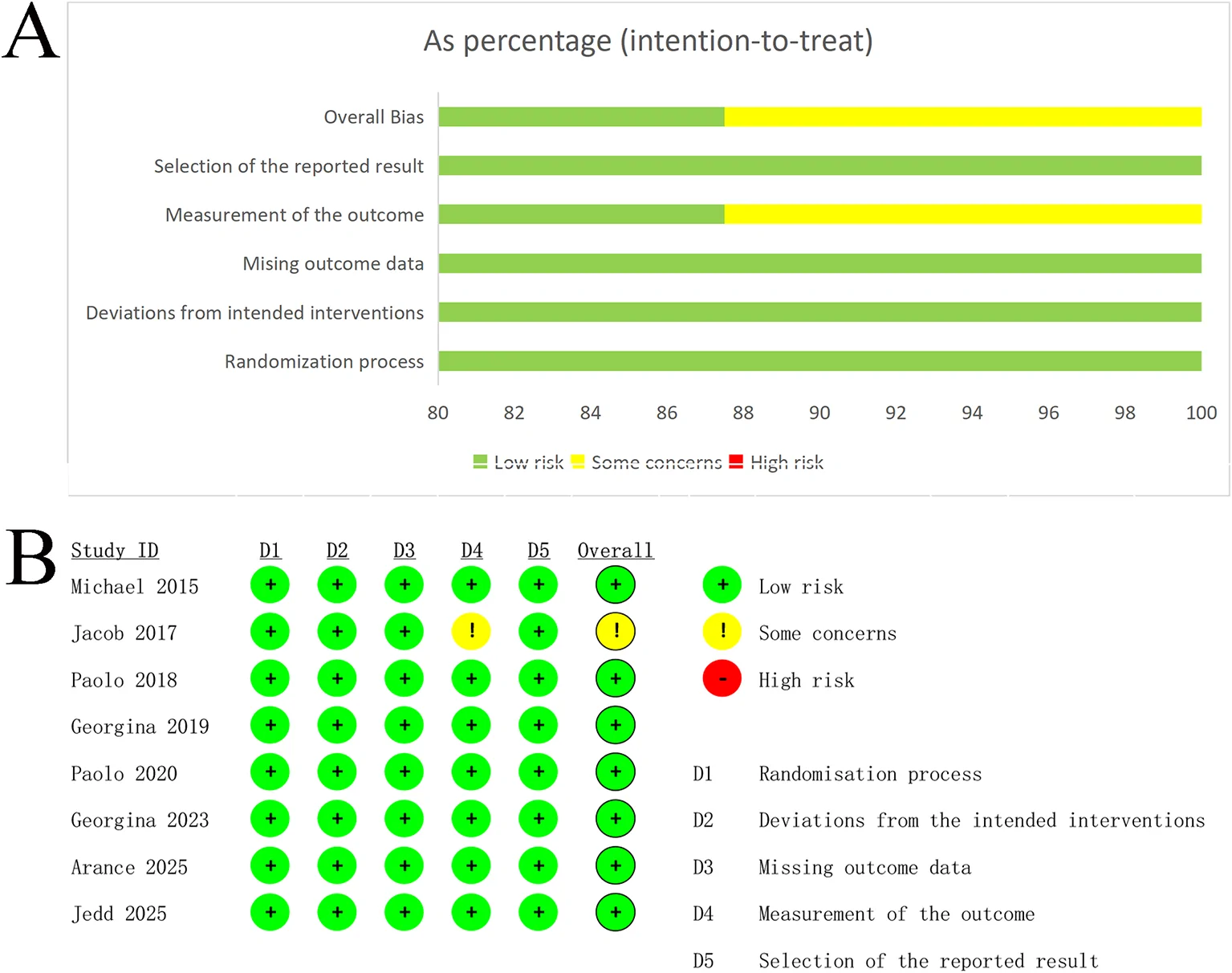
**(A)** Summary plot of evidence for risk of bias assessed by ROB2; **(B)** Proportion plot of risk of bias assessment.

### 3.4 NMA results for OS

In total, seven studies reported OS, involving 3,309 participants and nine interventions (**Figure 3A**). Convergence was assessed using Gelman–Rubin diagnostics and graphical inspection (**Supplementary Figure 1**). As no closed loops were formed in the network, inconsistency testing was not performed. The NMA showed that, relative to the commonly used clinical agent ipilimumab, pembrolizumab (HR = 0.70, 95% CrI: 0.52 to 0.94), nivolumab (HR = 0.61, 95% CrI: 0.49 to 0.77), nivolumab plus relatlimab (HR = 0.51, 95% CrI: 0.35 to 0.72), and nivolumab plus ipilimumab (HR = 0.58, 95% CrI: 0.46 to 0.73) significantly improved OS. Conversely, dacarbazine, epacadostat plus pembrolizumab, ipilimumab 10 mg/kg, and lenvatinib plus pembrolizumab failed to show a survival benefit in OS relative to ipilimumab. In the primary analysis of the connected network for OS, nivolumab plus relatlimab had the highest SUCRA value (SUCRA = 93.94%), outperforming dacarbazine (HR = 0.38, 95% CrI: 0.26 to 0.55), ipilimumab 10 mg/kg (HR = 0.56, 95% CrI: 0.37 to 0.85), and lenvatinib plus pembrolizumab (HR = 0.55, 95% CrI: 0.32 to 0.94). Nivolumab plus ipilimumab ranked second for prolonging OS (SUCRA = 83.06%), showing superiority over dacarbazine (HR = 0.44, 95% CrI: 0.29 to 0.66) and ipilimumab 10 mg/kg (HR = 0.64, 95% CrI: 0.47 to 0.88). Moreover, pembrolizumab demonstrated significant improvements over dacarbazine (HR = 0.53, 95% CrI: 0.34 to 0.83). Meanwhile, nivolumab showed more promising efficacy relative to dacarbazine (HR = 0.46, 95% CrI: 0.36 to 0.59) and relative to ipilimumab 10 mg/kg (HR = 0.68, 95% CrI: 0.50 to 0.93) (**Supplementary Table 3**). In the supplementary sensitivity analysis using a random-effects model for OS, the posterior median of the between-study standard deviation τ was 0.386, with a 95% CrIs of 0.021 to 0.757. The DIC values for the fixed-effects and random-effects models were 16.01 and 16.10, respectively. The difference in DIC was small, indicating no clear preference for either model. The SUCRA ranking presented above is for exploratory purposes only; the order of the ranking does not establish that the combination of nivolumab and relatlimab is superior to the combination of nivolumab and ipilimumab.

**Figure 3:**
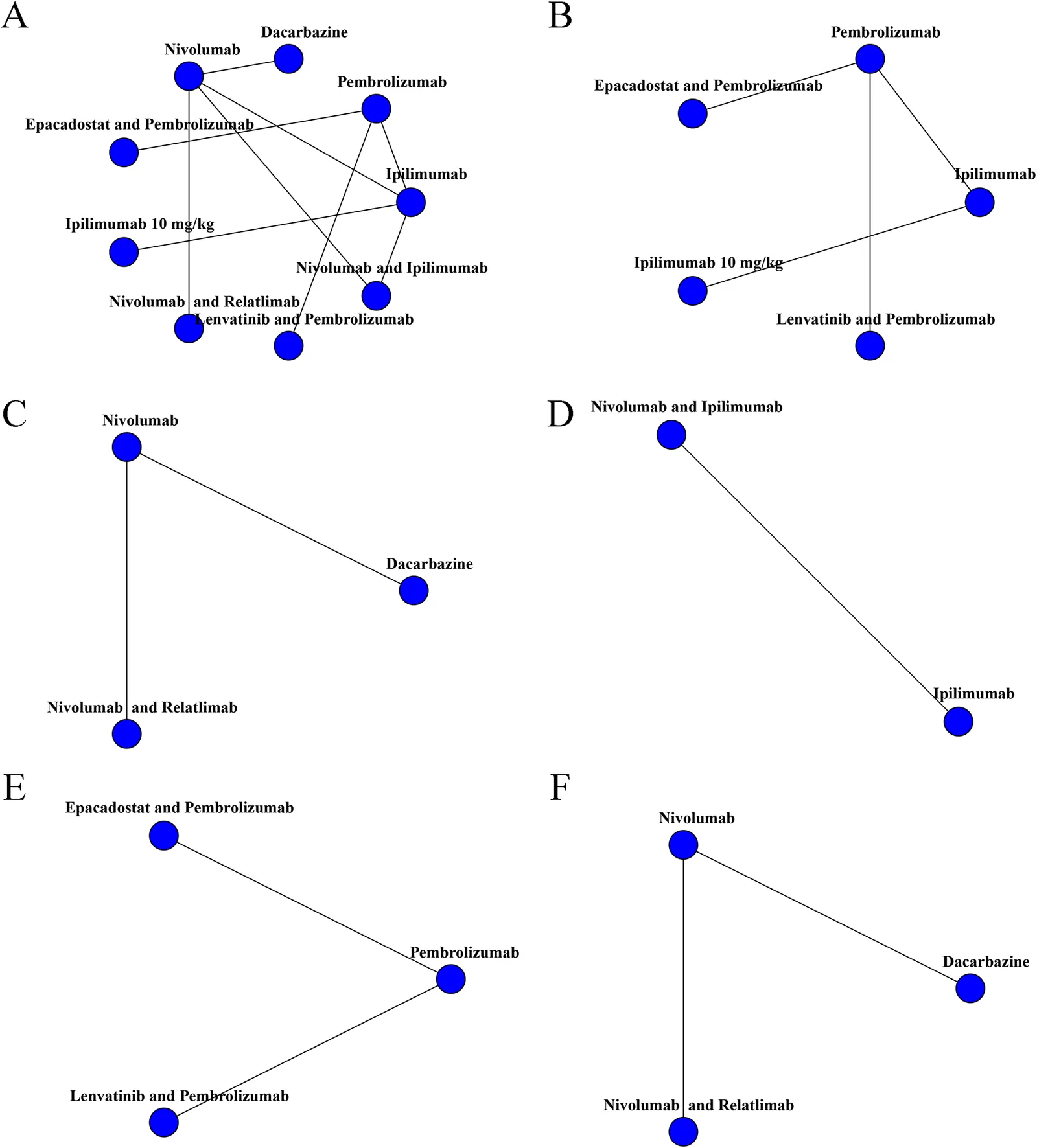
**(A)** Network plot of OS; **(B)** Network plot of OS after excluding Wolchok 2025 (part 1/2); **(C)** Network plot of OS after excluding Wolchok 2025 (part 2/2); **(D)** Network plot of PFS (part 1/3); **(E)** Network plot of PFS (part 2/3); **(F)** Network plot of PFS (part 3/3).

Further review of the original studies revealed that most trials reported follow-up periods of 2–6 years, whereas Jedd D. Wolchok (22) reported follow-up periods of 10 years. We therefore conducted an additional analysis after excluding the study. After exclusion, the network structure became disconnected. Therefore, two separate NMAs were performed (**Figures 3B, C**). In the NMA involving ipilimumab, pembrolizumab, epacadostat plus pembrolizumab, ipilimumab 10 mg/kg, and lenvatinib plus pembrolizumab, the league table and SUCRA values indicated that pembrolizumab was superior to ipilimumab (HR = 0.7, 95% CrI: 0.52 to 0.94) and lenvatinib plus pembrolizumab (HR = 0.76, 95% CrI: 0.58 to 0.99). In the NMA involving dacarbazine, nivolumab, and nivolumab plus relatlimab, nivolumab was superior to dacarbazine (HR = 0.46, 95% CrI: 0.36 to 0.59). Meanwhile, nivolumab plus relatlimab was superior to dacarbazine (HR = 0.38, 95% CrI: 0.26 to 0.55) (**Supplementary Tables 4**, **5**). Convergence diagnostics for the two disconnected components of the OS network are presented in **Supplementary Figures 2** and **3**. These results apply only to each connected component individually and do not support treatment comparisons or a unified ranking across the two components. Therefore, these analyses cannot fully validate the robustness of all comparisons and rankings in the original OS network.

### 3.5 NMA results for PFS

In total, 5 studies reported PFS, involving 1,784 participants and 8 interventions (**Figures 3D–F**). Convergence diagnostics for the three PFS network components are presented in **Supplementary Figures 4**-**6**. Given that no closed loops were formed in the network, inconsistency testing was not performed. Owing to the disconnected network structure, three separate NMAs were conducted (**Figures 3D–F**). In the NMA involving dacarbazine, nivolumab, and nivolumab plus relatlimab, relative to dacarbazine, nivolumab (HR = 0.42, 95% CrI: 0.33 to 0.53) and nivolumab plus relatlimab (HR = 0.33, 95% CrI: 0.23 to 0.46) significantly improved PFS. In a three-regimen connected network comprising dacarbazine, nivolumab, and the combination of nivolumab and relatlimab, the combination of nivolumab and relatlimab was associated with a lower risk of disease progression or death compared with nivolumab alone (HR = 0.78, 95% CrI: 0.61 to 0.99). The combination also had the highest SUCRA value (98.86%) among the three regimens in this network. This ranking does not apply to other components of the PFS network and cannot be used to compare this regimen with treatments in other components, such as the combination of nivolumab and ipilimumab. No significant differences were observed in the remaining two NMAs (one involving ipilimumab and nivolumab plus ipilimumab; the other involving pembrolizumab, epacadostat plus pembrolizumab, and lenvatinib plus pembrolizumab) (Details are provided in **Supplementary Tables 6**-**8**).

### 3.6 ORR

Although several included RCTs reported ORR, the data available for the BRAF wild-type population did not form a connected network covering all relevant treatment strategies. Therefore, a unified NMA could not be conducted. The results presented here are limited to two treatment comparisons (15, 20). These comparisons are shown in **Supplementary Figures 7** and **8**: nivolumab plus ipilimumab versus ipilimumab alone (RR = 12.84, 95% CrI: 0.42 to 371.13), and nivolumab plus relatlimab versus nivolumab alone (RR = 9.64, 95% CrI: 0.38 to 245.08). The low number of objective response events in these comparisons resulted in unstable effect estimates and wide CrIs, indicating considerable uncertainty. Therefore, these findings should be regarded as exploratory results, and no definitive conclusions regarding the relative efficacy of treatment regimens should be drawn from them. The fact that the CrIs include the null value should not be interpreted as evidence of equivalent efficacy.

## 4 Discussion

This research employs an NMA to systematically evaluate the efficacy of diverse systemic treatment strategies for individuals with BRAF wild-type metastatic unresectable melanoma. These findings indicate that pembrolizumab, nivolumab, nivolumab plus relatlimab, and nivolumab plus ipilimumab were associated with improved OS compared with ipilimumab. Nivolumab plus relatlimab had the highest SUCRA values in the primary OS network and in the corresponding PFS connected network (OS: SUCRA = 93.94%, PFS: SUCRA = 98.86%). These rankings are exploratory only and do not support comparisons across disconnected networks, nor do they establish that this regimen is superior to all other treatments.

Nivolumab and pembrolizumab target PD-1, whereas nivolumab plus relatlimab blocks both PD-1 and LAG-3 (23, 24). These mechanisms provide a biological basis for the treatment strategies under evaluation, but they do not in themselves prove that one regimen is superior to another in terms of efficacy or safety. The validity of the indirect comparisons in this study depends on the transitivity assumption. The distribution of potential effect modifiers across different treatment comparisons should be sufficiently similar. Limiting the study population solely to patients with BRAF wild-type metastatic unresectable melanoma does not guarantee that this assumption holds true. Previous systemic therapy, performance status, LDH levels, brain metastases, and disease burden are all relevant characteristics that must be considered. In particular, some of the included trials did not report data separately for patients who had received no prior systemic therapy. Therefore, uncertainty remains regarding differences in treatment lines among the various treatment comparisons. Additionally, there were variations in follow-up duration across the studies. After the exclusion of a study with a follow-up period of 10 years, the OS network became disconnected, indicating that this study played a crucial role in linking different treatment comparisons. However, this disconnection also limited the ability of this sensitivity analysis to assess the robustness of the complete network results. Given these factors, the effect estimates and treatment rankings from indirect comparisons should be interpreted with caution.

In patients with BRAF-mutant melanoma, whose tumors are oncogene-driven and for whom targeted therapy is available, the preferred sequence for most patients should be nivolumab plus ipilimumab, with subsequent BRAF/MEK inhibition if necessary. Sequential immunotherapy and targeted therapy provide clinically meaningful survival benefits for patients with BRAF V600-mutant melanoma (25, 26). For patients with BRAF V600 wild-type metastatic unresectable melanoma, targeted therapies directed against the BRAF V600 mutation are not applicable. Immunotherapy is therefore an important systemic treatment option. However, BRAF mutation status alone cannot be used to directly predict the extent of benefit from immunotherapy. This study evaluates only the relative efficacy of different treatment regimens in the BRAF wild-type population and does not compare the efficacy of these regimens with that observed in the BRAF-mutated population.

This NMA showed that, compared with ipilimumab monotherapy, the combination of nivolumab and ipilimumab was associated with improved OS (HR = 0.58). Nivolumab plus relatlimab, nivolumab monotherapy, and pembrolizumab monotherapy all significantly improved OS compared with ipilimumab. In contrast, current evidence has not demonstrated that epacadostat plus pembrolizumab improved OS compared with ipilimumab. However, this observation should not be interpreted as evidence of no benefit. These findings suggest that OS results should be interpreted in the context of specific treatment regimens and their comparators.

Accordingly, the optimal combination strategies for immunotherapy remain under active investigation, particularly across distinct subtypes of melanoma. Although certain immunotherapy combinations possess theoretical potential for enhanced efficacy, their actual clinical benefits may vary due to interpatient variability, characteristics of the tumor microenvironment, and tolerability for treatment. Consequently, for older or immunocompromised patients who are unable to tolerate severe toxicities, monotherapy may represent a more appropriate therapeutic option.

With the advancement of research in immunotherapy, particularly the combined application of ICIs with novel immunologic targets (e.g., LAG-3 inhibitors and anti-angiogenic agents), more therapeutic options and survival benefits might become available for individuals with BRAF wild-type unresectable metastatic melanoma. Future studies should further investigate the differential efficacy of immunotherapy across distinct molecular subtypes of melanoma, especially regarding the biological and clinical differences between individuals with BRAF-mutant melanoma and those with BRAF wild-type melanoma. Through more precise molecular characterization, increasingly personalized treatment strategies can be offered.

This research has several limitations. First, the number of included studies in the NMA is relatively limited. Specifically, concerning comparisons of immunotherapy combinations, the available evidence is insufficient to draw broader conclusions. Second, clinical data for certain treatment regimens are sparse, and follow-up durations vary across studies, which might have contributed to increased heterogeneity in the analysis. Third, though the treatment regimens were compared utilizing a Bayesian framework, the absence of closed loops for certain treatment combinations precluded formal assessment of inconsistency between direct and indirect evidence. Finally, the study populations were predominantly concentrated in Europe, North America, and specific Asian countries. The lack of multicenter data from other geographic regions might constrain the generalizability of our findings. This study aimed to fully utilize the available evidence to explore systemic treatment regimens that may offer benefits and to estimate relative efficacy and treatment sequencing under the fixed-effects assumption. However, since this model assumes that the true effect of a given treatment comparison is consistent across studies, and some RCTs did not report data specifically for patients who had received no prior systemic therapy. The uncertainty arising from heterogeneity among studies and differences across treatment lines may not have been fully accounted for. These findings should therefore be considered exploratory and need to be validated in further high-quality studies.

This study provides evidence regarding the comparative efficacy of different systemic treatment regimens for BRAF wild-type metastatic unresectable melanoma. The primary analysis of OS supports a survival benefit for some regimens compared with ipilimumab. However, the current evidence is insufficient to identify a single regimen that is superior across all relevant treatment options. In particular, the lack of network connectivity for PFS limits a comprehensive comparison across regimens. All SUCRA rankings should be interpreted within the context of the corresponding outcomes and connected networks. They should be treated as exploratory findings for generating research hypotheses and should not be used alone as a basis for changing current standard of care. Future direct head-to-head RCTs are needed to further clarify the relative benefits and risks of the relevant treatment strategies.

## Data Availability

The original contributions presented in the study are included in the article/**Supplementary Material**. Further inquiries can be directed to the corresponding author.
