# Supplementary material for: Systemic treatment strategies for BRAF wild-type metastatic unresectable melanoma: a network meta-analysis
Source: Front Oncol. 2026 Oct 2;16:1967814. doi: 10.3389/fonc.2026.1967814 (PMC13672231; doi:10.3389/fonc.2026.1967814)
Supplement: Supplementary file 1 [file Table1.docx]

# Supplementary Materials

**Supplementary Table 1** Record of search results.

| Pubmed | | |
| --- | --- | --- |
| # | Query | Results |
| #1 | Neoplasm Metastasis[MeSH Terms] | 238523 |
| #2 | "Neoplasm Metastasis"[Title/Abstract] OR "Metastase"[Title/Abstract] OR "Metastases"[Title/Abstract] OR "Metastasis"[Title/Abstract] OR "Neoplasm Metastases"[Title/Abstract] OR "cancer cell dissemination"[Title/Abstract] OR "cancer cell metastasis"[Title/Abstract] OR "cancer cell spread"[Title/Abstract] OR "cancer dissemination"[Title/Abstract] OR "cancer metastasis"[Title/Abstract] OR "cancer spread"[Title/Abstract] OR "carcinoma metastasis"[Title/Abstract] OR "disseminated tumor cell"[Title/Abstract] OR "disseminated tumour cell"[Title/Abstract] OR "metastasic type"[Title/Abstract] OR "metastasis formation"[Title/Abstract] OR "metastatic cancer"[Title/Abstract] OR "metastatic cancers"[Title/Abstract] OR "metastatic carcinoma"[Title/Abstract] OR "metastatic carcinomas"[Title/Abstract] OR "metastatic disease"[Title/Abstract] OR "metastatic tumor"[Title/Abstract] OR "metastatic tumors"[Title/Abstract] OR "metastatic tumour"[Title/Abstract] OR "metastatic tumours"[Title/Abstract] OR "neoplastic cell dissemination"[Title/Abstract] OR "sarcoma metastasis"[Title/Abstract] OR "secondary cancer"[Title/Abstract] OR "secondary carcinoma"[Title/Abstract] OR "tumor dissemination"[Title/Abstract] OR "tumor metastasis"[Title/Abstract] OR "tumor migration"[Title/Abstract] OR "tumor spread"[Title/Abstract] OR "tumour dissemination"[Title/Abstract] OR "tumour metastasis"[Title/Abstract] OR "tumour migration"[Title/Abstract] OR "tumour spread"[Title/Abstract] OR "advanced"[Title/Abstract] OR "unresectable"[Title/Abstract] OR "metastatic"[Title/Abstract] | 1399628 |
| #3 | Melanoma[MeSH Terms] | 119644 |
| #4 | "Melanoma"[Title/Abstract] OR "Melanomas"[Title/Abstract] OR "Malignant Melanoma"[Title/Abstract] OR "Malignant Melanomas"[Title/Abstract] OR "fortner melanoma"[Title/Abstract] OR "malignant melanoma"[Title/Abstract] OR "melano carcinoma"[Title/Abstract] OR "melano sarcoma"[Title/Abstract] OR "melanoblastoma"[Title/Abstract] OR "melanocarcinoma"[Title/Abstract] OR "melanocytic malignancies"[Title/Abstract] OR "melanocytic malignancy"[Title/Abstract] OR "melanomalignoma"[Title/Abstract] OR "melanosarcoma"[Title/Abstract] OR "melanotic carcinoma"[Title/Abstract] OR "pigmentary cancer"[Title/Abstract] | 162144 |
| #5 | Randomized Controlled Trial[Publication Type] | 670526 |
| #6 | "Randomized Controlled Trial"[Title/Abstract] OR "Controlled Clinical Trial"[Title/Abstract] OR "Clinical Trial"[Title/Abstract] OR "Clinical Study"[Title/Abstract] OR "RCT"[Title/Abstract] OR "randomised controlled study"[Title/Abstract] OR "randomised controlled trial"[Title/Abstract] OR "randomized controlled study"[Title/Abstract] | 548124 |
| #7 | (#1 OR #2) AND (#3 OR #4) AND (#5 OR #6) | 2258 |

| Embase | | |
| --- | --- | --- |
| # | Query | Results |
| #1 | 'metastasis'/exp | 1027172 |
| #2 | 'neoplasm metastasis':ti,ab,kw OR 'metastase':ti,ab,kw OR 'metastases':ti,ab,kw OR 'metastasis':ti,ab,kw OR 'neoplasm metastases':ti,ab,kw OR 'cancer cell dissemination':ti,ab,kw OR 'cancer cell metastasis':ti,ab,kw OR 'cancer cell spread':ti,ab,kw OR 'cancer dissemination':ti,ab,kw OR 'cancer metastasis':ti,ab,kw OR 'cancer spread':ti,ab,kw OR 'carcinoma metastasis':ti,ab,kw OR 'disseminated tumor cell':ti,ab,kw OR 'disseminated tumour cell':ti,ab,kw OR 'metastasic type':ti,ab,kw OR 'metastasis formation':ti,ab,kw OR 'metastatic cancer':ti,ab,kw OR 'metastatic cancers':ti,ab,kw OR 'metastatic carcinoma':ti,ab,kw OR 'metastatic carcinomas':ti,ab,kw OR 'metastatic disease':ti,ab,kw OR 'metastatic tumor':ti,ab,kw OR 'metastatic tumors':ti,ab,kw OR 'metastatic tumour':ti,ab,kw OR 'metastatic tumours':ti,ab,kw OR 'neoplastic cell dissemination':ti,ab,kw OR 'sarcoma metastasis':ti,ab,kw OR 'secondary cancer':ti,ab,kw OR 'secondary carcinoma':ti,ab,kw OR 'tumor dissemination':ti,ab,kw OR 'tumor metastasis':ti,ab,kw OR 'tumor migration':ti,ab,kw OR 'tumor spread':ti,ab,kw OR 'tumour dissemination':ti,ab,kw OR 'tumour metastasis':ti,ab,kw OR 'tumour migration':ti,ab,kw OR 'tumour spread':ti,ab,kw OR 'advanced':ti,ab,kw OR 'unresectable':ti,ab,kw OR 'metastatic':ti,ab,kw | 2135368 |
| #3 | 'melanoma'/exp | 248591 |
| #4 | 'melanoma':ti,ab,kw OR 'melanomas':ti,ab,kw OR 'malignant melanoma':ti,ab,kw OR 'malignant melanomas':ti,ab,kw OR 'fortner melanoma':ti,ab,kw OR 'malignant melanoma':ti,ab,kw OR 'melano carcinoma':ti,ab,kw OR 'melano sarcoma':ti,ab,kw OR 'melanoblastoma':ti,ab,kw OR 'melanocarcinoma':ti,ab,kw OR 'melanocytic malignancies':ti,ab,kw OR 'melanocytic malignancy':ti,ab,kw OR 'melanomalignoma':ti,ab,kw OR 'melanosarcoma':ti,ab,kw OR 'melanotic carcinoma':ti,ab,kw OR 'pigmentary cancer':ti,ab,kw | 240141 |
| #5 | 'randomized controlled trial'/exp | 1240582 |
| #6 | 'randomized controlled trial':ti,ab,kw OR 'controlled clinical trial':ti,ab,kw OR 'clinical trial':ti,ab,kw OR 'clinical study':ti,ab,kw OR 'rct':ti,ab,kw OR 'randomised controlled study':ti,ab,kw OR 'randomised controlled trial':ti,ab,kw OR 'randomized controlled study':ti,ab,kw | 952865 |
| #7 | (#1 OR #2) AND (#3 OR #4) AND (#5 OR #6) | 12299 |

| Cochrane Library | | |
| --- | --- | --- |
| # | Query | Results |
| #1 | MeSH descriptor: [Neoplasm Metastasis] explode all trees | 8061 |
| #2 | ('Neoplasm Metastasis' OR 'Metastase' OR 'Metastases' OR 'Metastasis' OR 'Neoplasm Metastases' OR 'cancer cell dissemination' OR 'cancer cell metastasis' OR 'cancer cell spread' OR 'cancer dissemination' OR 'cancer metastasis' OR 'cancer spread' OR 'carcinoma metastasis' OR 'disseminated tumor cell' OR 'disseminated tumour cell' OR 'metastasic type' OR 'metastasis formation' OR 'metastatic cancer' OR 'metastatic cancers' OR 'metastatic carcinoma' OR 'metastatic carcinomas' OR 'metastatic disease' OR 'metastatic tumor' OR 'metastatic tumors' OR 'metastatic tumour' OR 'metastatic tumours' OR 'neoplastic cell dissemination' OR 'sarcoma metastasis' OR 'secondary cancer' OR 'secondary carcinoma' OR 'tumor dissemination' OR 'tumor metastasis' OR 'tumor migration' OR 'tumor spread' OR 'tumour dissemination' OR 'tumour metastasis' OR 'tumour migration' OR 'tumour spread' OR 'advanced' OR 'unresectable' OR 'metastatic'):ti,ab,kw | 169638 |
| #3 | MeSH descriptor: [Melanoma] explode all trees | 2909 |
| #4 | ('Melanoma' OR 'Melanomas' OR 'Malignant Melanoma' OR 'Malignant Melanomas' OR 'fortner melanoma' OR 'malignant melanoma' OR 'melano carcinoma' OR 'melano sarcoma' OR 'melanoblastoma' OR 'melanocarcinoma' OR 'melanocytic malignancies' OR 'melanocytic malignancy' OR 'melanomalignoma' OR 'melanosarcoma' OR 'melanotic carcinoma' OR 'pigmentary cancer'):ti,ab,kw | 7582 |
| #5 | MeSH descriptor: [Randomized Controlled Trial] explode all trees | 34 |
| #6 | ('Randomized Controlled Trial' OR 'Controlled Clinical Trial' OR 'Clinical Trial' OR 'Clinical Study' OR 'RCT' OR 'randomised controlled study' OR 'randomised controlled trial' OR 'randomized controlled study'):ti,ab,kw | 1377166 |
| #7 | (#1 OR #2) AND (#3 OR #4) AND (#5 OR #6) | 3526 |

| Web of Science | | |
| --- | --- | --- |
| # | Query | Results |
| #10 | TS=("Neoplasm Metastasis" OR "Metastase" OR "Metastases" OR "Metastasis" OR "Neoplasm Metastases" OR "cancer cell dissemination" OR "cancer cell metastasis" OR "cancer cell spread" OR "cancer dissemination" OR "cancer metastasis" OR "cancer spread" OR "carcinoma metastasis" OR "disseminated tumor cell" OR "disseminated tumour cell" OR "metastasic type" OR "metastasis formation" OR "metastatic cancer" OR "metastatic cancers" OR "metastatic carcinoma" OR "metastatic carcinomas" OR "metastatic disease" OR "metastatic tumor" OR "metastatic tumors" OR "metastatic tumour" OR "metastatic tumours" OR "neoplastic cell dissemination" OR "sarcoma metastasis" OR "secondary cancer" OR "secondary carcinoma" OR "tumor dissemination" OR "tumor metastasis" OR "tumor migration" OR "tumor spread" OR "tumour dissemination" OR "tumour metastasis" OR "tumour migration" OR "tumour spread" OR "advanced" OR "unresectable" OR "metastatic") | 2217666 |
| #12 | TS=("Melanoma" OR "Melanomas" OR "Malignant Melanoma" OR "Malignant Melanomas" OR "fortner melanoma" OR "malignant melanoma" OR "melano carcinoma" OR "melano sarcoma" OR "melanoblastoma" OR "melanocarcinoma" OR "melanocytic malignancies" OR "melanocytic malignancy" OR "melanomalignoma" OR "melanosarcoma" OR "melanotic carcinoma" OR "pigmentary cancer") | 221382 |
| #13 | TS=("Randomized Controlled Trial" OR "Controlled Clinical Trial" OR "Clinical Trial" OR "Clinical Study" OR "RCT" OR "randomised controlled study" OR "randomised controlled trial" OR "randomized controlled study") | 688060 |
| #15 | #10 AND #12 AND #13 | 2455 |

**Supplementary Table 2** Basic features of included studies

| General Information | | | | Demographic information of population | | | | | | | | | Intervention | | | | | | Control | | | | | | O | Study Type (focus on registration number) |
| --- | --- | --- | --- | --- | --- | --- | --- | --- | --- | --- | --- | --- | --- | --- | --- | --- | --- | --- | --- | --- | --- | --- | --- | --- | --- | --- |
| The surname of the first author | Publication Year | Rescreening status | Country | Sample Size | Age | Gender | Severity of disease | M stage, | ECOG | LDH | brain metastases | PD-L1 | Name of interventions |  | Dosage | Route of administration | Frequency | Duration | Name of interventions in control group |  | Dosage | Route of administration | Frequency | Duration | Outcome |  |
| Michael A. Postow | 2015 | Eligible | the United States and France | 109 | Nivolumab plus Ipilimumab 66（27-87） Ipilimumab 69（46-80） | 71 /38 | Eligible patients had histologically confirmed, unresectable, previously untreated stage III or IV melanoma with measurable disease. Other inclu- sion criteria included a known BRAF V600 muta- tion status, an Eastern Cooperative Oncology Group performance-status score of 0 or 1 (on a scale of 0 to 5, with 0 indicating no symptoms and high- er scores indicating greater disability), and the availability of tumor tissue from a metastatic or unresectable site for immunohistochemical assess- ment of PD-1 ligand (PD-L1) expression. Key ex- clusion criteria were active brain metastases, uveal melanoma, and serious autoimmune disease. | M0 Nivolumab plus Ipilimumab 6 (8) Ipilimumab 5 (14) M1a Nivolumab plus Ipilimumab 9 (12) Ipilimumab 7 (19) M1b Nivolumab plus Ipilimumab 22 (31) Ipilimumab 8 (22) M1c Nivolumab plus Ipilimumab 34 (47) Ipilimumab 16 (43) Not reported Nivolumab plus Ipilimumab 1 (1) Ipilimumab 1 (3) | 0 Nivolumab plus Ipilimumab 62 (86) Ipilimumab 30 (81) 1 Nivolumab plus Ipilimumab 9 (12) Ipilimumab 7 (19) ≥2 Nivolumab plus Ipilimumab 1 (1) Ipilimumab 0 | ≤ULN Nivolumab plus Ipilimumab 57 (79) Ipilimumab 30 (81) >ULN Nivolumab plus Ipilimumab 15 (21) Ipilimumab 7 (19) ≤2× ULN Nivolumab plus Ipilimumab 69 (96) Ipilimumab 36 (97) >2× ULN Nivolumab plus Ipilimumab 3 (4) Ipilimumab 1 (3) | Yes Nivolumab plus Ipilimumab 4 (6) Ipilimumab 0 No Nivolumab plus Ipilimumab 67 (93) Ipilimumab 37 (100) | NA | nivolumab plus ipilimumab | 72 | Nivolumab at a dose of 1 mg per kilogram of body weight; Ipilimumab at a dose of 3 mg per kilogram;  After the fourth dose of both drugs, ipilimumab is discontinued, and the regimen transitions to monotherapy with nivolumab administered intravenously over 60 minutes at a dose of 3 mg per kilogram of body weight during the maintenance phase. | Intravenous injection | Nivolumab and ipilimumab are administered every three weeks for a total of four doses; nivolumab is then given as monotherapy every two weeks. | 15 month | ipilimumab alone | 37 | Placebo at a dose of 1 mg per kilogram of body weight; Ipilimumab at 3 mg per kilogram  After the fourth dose of both agents, ipilimumab is discontinued, and monotherapy with placebo is administered intravenously over 60 minutes at a dose of 3 mg per kilogram of body weight during the maintenance phase. | Intravenous injection | Placebo and ipilimumab every 3 weeks for a total of four doses. Placebo is administered as monotherapy every two weeks. | 15 month | pfs ：Nivolumab and Ipilimumab versus Ipilimumab Hazard ratio, 0.40 (95% CI, 0.23–0.68) P<0.001  Objective Response Rate : Nivolumab and Ipilimumab versus Ipilimumab OR=12.96 (95% CI, 3.91–54.49)   Nivolumab plus Ipilimumab (N = 94): Adverse events 86, Grade 3-4 events 51 Ipilimumab monotherapy (N = 46): Adverse events 43, Grade 3-4 events 11 | NCT01927419. |
| Jacob Schachter | 2017 | Eligible | 16 countries (Australia, Austria, Belgium, Canada, Chile, Colombia, France, Germany, Israel, Netherlands, New Zealand, Norway, Spain, Sweden, UK, and USA) | 834 (525 with BRAF wild-type) | Pembrolizumab every 2 weeks 61 (18–89)  Pembrolizumab every 3 weeks 63 (22–89)  Ipilimumab 62 (18–88) | 497 / 337 | Eligible patients were aged at least 18 years, with an Eastern Cooperative Oncology Group (ECOG) perfor­ mance status of 0 or 1, at least one measurable lesion per Response Evaluation Criteria In Solid Tumors version 1.1 (RECIST v1.1), unresectable stage III or IV melanoma (excluding ocular melanoma), and up to one previous systemic therapy (excluding anti-CTLA-4, PD-1, or PD-L1 agents). | M0 Pembrolizumab every 2 weeks 9 (3%) Pembrolizumab every 3 weeks 8 (3%) Ipilimumab 13 (5%) M1 Pembrolizumab 6 (2%) Pembrolizumab every 3 weeks 4 (1%) Ipilimumab 5 (2%) M1a Pembrolizumab every 2 weeks 21 (8%) Pembrolizumab every 3 weeks 35 (13%) Ipilimumab 30 (11%) M1b Pembrolizumab every 2 weeks 64 (23%) Pembrolizumab every 3 weeks 41 (15%) Ipilimumab 52 (19%) M1c Pembrolizumab every 2 weeks179 (64%) Pembrolizumab every 3 weeks189 (68%) Ipilimumab178 (64%) | 0 Pembrolizumab every 2 weeks 196 (70%) Pembrolizumab every 3 weeks 18(68%） Ipilimumab 188 (68%) 1 Pembrolizumab every 2 weeks 83 (30%) Pembrolizumab every 3 weeks 88 (32%) Ipilimumab 90 (32%) | Normal Pembrolizumab every 2 weeks 194 (70%) Pembrolizumab every 3 weeks 175 (63%) Ipilimumab 178 (64%) Eleva embrolizumab every 2 weeks 81 (29%) Pembrolizumab every 3 weeks 98 (35%) Ipilimumab 91 (33%) Missing Pembrolizumab every 2 weeks 4(1%) Pembrolizumab every 3 weeks 4 (1%) Ipilimumab 9 (3%) | Yes Pembrolizumab every 2 weeks 24(9%) Pembrolizumab every 3 weeks 27(10%) Ipilimumab 29 (10%) No Pembrolizumab every 2 weeks 252 (90%) Pembrolizumab every 3 weeks 248 (90%) Ipilimumab 248 (89%) Missing Pembrolizumab every 2 weeks 3 (1%) Pembrolizumab every 3 weeks 2 (1%) Ipilimumab 1 (<1%) | Positive Pembrolizumab every 2 weeks 225 (81%) Pembrolizumab every 3 weeks 221 (80%) Ipilimumab 225 (81%) Negative Pembrolizumab every 2 weeks 49 (18%) Pembrolizumab every 3 weeks 54 (20%) Ipilimumab 47 (17%) Unknown Pembrolizumab every 2 weeks 5 (2%) Pembrolizumab every 3 weeks 2 (1%) Ipilimumab 6 (2%) | Pembrolizumab | 355 (177 every two weeks, 128 every three weeks) | 10 mg/kg | Intravenous injection | Every 2-3 weeks | 26 month | Ipilimumab | 170 | 3 mg/kg | Intravenous injection | Every 3 weeks | 26 month | os：  Pembrolizumab versus ipilimumab 0.699（0.4966-0.9014）  Pembrolizumab Every 2 weeks: n=278, AEs 229 (grade 3-4: 47) Pembrolizumab Every 3 weeks: n=277, AEs 213 (grade 3-4: 46) Ipilimumab n=256, AEs 190 (grade 3-4: 50) | (KEYNOTE-006) NCT01866319 |
| Paolo A. Ascierto | 2018 | Eligible | Europe, Israel, Australia, Canada, and South America | 418 | Nivolumab 64 (18-86)  Dacarbazine 66 (25-87) | 246 / 172 | In brief, eligible patients were 18 years or older with an Eastern Cooperative Oncology Groupperformance status of0or 1 andhad histologically con- firmed unresectable previously untreated stage III or IV mela- noma but without a BRAF mutation. | M0/M1a/M1b Nivolumab 82 (39.0) Dacarbazine 81 (38.9) M1c Nivolumab 128 (61.0) Dacarbazine 127 (61.1) | 0 Nivolumab 148 (70.5) Dacarbazine 121 (58.2) 1 Nivolumab 60 (28.6) Dacarbazine 84 (40.4) | ≤ULN Nivolumab 120 (57.1) Dacarbazine 125 (60.1) >ULN Nivolumab 79 (37.6) Dacarbazine 74 (35.6) Not reported Nivolumab 11 (5.2) Dacarbazine 9 (4.3) | NA | Positive, ≥5% expression Nivolumab 59 (28.1) Dacarbazine 61 (29.3) Negative or indeterminate Nivolumab 151 (71.9) Dacarbazine 147 (70.7) | nivolumab | 210 | Nivolumab 3 mg/kg, Dacarbazine-matching placebo 1000 mg/m² | Intravenous injection | Nivolumab (every 2 weeks)  Dacarbazine-matching placebo (every 3 weeks) | 51 month | dacarbazine | 208 | Dacarbazine 1000 mg/m², Nivolumab-matching placebo 3 mg/kg， | Intravenous injection | Dacarbazine every 3 weeks, Nivolumab-matching placebo every 2 weeks | 51 month | os： nivolumab vs dacarbazine hazard ratio, 0.46; 95% CI, 0.36-0.59; P < .001  pfs： nivolumab vs dacarbazine (hazard ratio, 0.42; 95% CI, 0.33-0.53; P < .001)  (follow up 51M) | The CheckMate 066NCT01721772 |
| Georgina V Long | 2019 | Eligible | 23 countries | 706（392 with BRAF wild-type） | Epacadostat plus pembrolizumab 64 (52−72)*  Placebo plus pembrolizumab 63 (53·5−72)* | 423 / 283 | Eligible participants were aged 18 years or older who had histologically or cytologically confirmed, unresectable stage III or stage IV melanoma not amenable to local therapy; an ECOG performance status of 0 or 1; a known BRAFV600 mutation status or had consented to BRAFV600 mutation testing during screening; measurable disease in accordance with sponsor-modified Response Evaluation Criteria in Solid Tumors (RECIST) version 1.1 (version 1.1 criteria, but with up to ten lesions and up to five lesions per organ [version 1.0 criteria] instead of up to five lesions and up to two lesions per organ [version 1.1 criteria]; cutaneous lesions and other superficial lesions were not deemed measurable lesions, but could be deemed non-target lesions); and no previous adjuvant therapy or treatment for advanced or metastatic disease, except for BRAF or MEK inhibitors (alone or in combination) for BRAFV600 mutant melanoma, previous adjuvant or neoadjuvant melanoma therapy if completed 4 weeks or longer before randomisation and all treatment- related adverse events either returned to baseline or stabilised, and previous adjuvant therapy containing immunotherapy (eg, interferon or anti-CTLA-4 therapy, excluding anti-PD1-based adjuvant therapy) if relapse did not occur during treatment or within 6 months of treatment discontinuation. | M0 Epacadostat plus pembrolizumab 14 (4%) pembrolizumab 16 (5%) M1a Epacadostat plus pembrolizumab 39 (11%) pembrolizumab 43 (12%) M1b Epacadostat plus pembrolizumab 73 (21%) pembrolizumab 79 (22%) M1c Epacadostat plus pembrolizumab 228 (64%) pembrolizumab 214 (61%) | 0 Epacadostat plus pembrolizumab 261 (74%) pembrolizumab 267 (76%) 1 Epacadostat plus pembrolizumab 93 (26%) pembrolizumab 85 (24%) | >ULN Epacadostat plus pembrolizumab 123 (35%) pembrolizumab 113 (32%) >ULN but <2 times ULN Epacadostat plus pembrolizumab 99 (28%) pembrolizumab 93 (26%) ≥2 times ULN Epacadostat plus pembrolizumab 24 (7%) pembrolizumab 20 (6%) | Epacadostat plus pembrolizumab 19 (5%) pembrolizumab 14 (4%) | Positive Epacadostat plus pembrolizumab 257 (73%) pembrolizumab 255 (72%) Negative Epacadostat plus pembrolizumab 97 (27%) pembrolizumab 97 (28%) | epacadostat plus pembrolizumab | 196 | epacadostat 100mg  pembrolizumab 200 mg | Epacadostat administered orally Pembrolizumab administered intravenously | Epacadostat twice daily, Pembrolizumab every 3 weeks | 18 month | placebo plus pembrolizumab | 196 | placebo100mg  pembrolizumab 200 mg | Placebo orally Pembrolizumab intravenously | Placebo twice daily Pembrolizumab every 3 weeks | 18 month | os： epacadostat plus pembrolizuma vs pembrolizumab   HR R(95% CI) 1·29 (0·90–1·84)    pfs： epacadostat plus pembrolizuma vs pembrolizumabHR R(95% CI) 1·02 (0·79–1·31) | ECHO-301/KEYNOTE-252(NCT02752074.) |
| Paolo Antonio Ascierto | 2020 | Eligible | 21 countries | 727（462 with BRAF wild-type） | Ipilimumab 10 mg/kg 62 (49–70)  Ipilimumab 3 mg/kg  62 (51–71) | 450 / 277 | Details of the study design and eligibility criteria have been described previously.11 Eligible patients were aged ≥18 years and had untreated or previously treated unre- sectable stage III/IV metastatic melanoma, an Eastern Cooperative Oncology Group performance status (ECOG PS) score of 0 or 1 and measurable disease within 28 days of the first dose of study treatment, based on modified WHO diagnostic criteria. Patients who had received prior therapy with BRAF inhibitors, CTLA-4 or PD-1 antago- nists or programmed death-­ligand 1 or CD137 agonists were excluded, as were patients with symptomatic brain metastases or brain metastases requiring treatment, a history of autoimmune disease or a diagnosis of primary ocular melanoma. | M0, M1a, or M1b Ipilimumab 10 mg/kg 136 (37%) Ipilimumab 3mg/kg 142 (39%) M1c without brain metastases Ipilimumab 10 mg/kg 164 (45%) Ipilimumab 3mg/kg 158 (44%) M1c with brain metastases Ipilimumab 10 mg/kg 65 (18%) Ipilimumab 3mg/kg 62 (17%) | 0 Ipilimumab 10 mg/kg 262 (72%) Ipilimumab 3 mg/kg 253 (70%) 1 Ipilimumab 10 mg/kg 103 (28%) Ipilimumab 3 mg/kg 109 (30%) | ≤ULN Ipilimumab 10 mg/kg 222 (61%) Ipilimumab 3 mg/kg 219 (60%) >ULN Ipilimumab 10 mg/kg 133 (36%) Ipilimumab 3 mg/kg 136 (38%) ≤2 × ULN Ipilimumab 10 mg/kg 321 (88%) Ipilimumab 3 mg/kg 306 (85%) >2 × ULN Ipilimumab 10 mg/kg 34 (9%) Ipilimumab 3 mg/kg 49 (14%) Not reported Ipilimumab 10 mg/kg 10 (3%)  Ipilimumab 3 mg/kg 7 (2%) | NA | NA | ipilimumab 10 mg/kg | 225 | 10 mg/kg | Intravenous injection | Every 3 weeks for a total of 4 doses | 65 month | ipilimumab 3 mg/kg | 237 | 3 mg/kg | Intravenous injection | Administered every 3 weeks for a total of 4 doses. | 65 month | os： ipilimumab 10 mg/kg vs ipilimumab 3 mg/kg  0.9（0.73-1.11）  Ipilimumab 10 mg/kg (n=364): Adverse events 347, Grade 3-5 events 228 Ipilimumab 3 mg/kg (n=362): Adverse events 341, Grade 3-5 events 196 | NCT01515189 |
| Georgina V. Long | 2023 | Eligible | North America, Central America, South America, Europe, Australia, and New Zealand. | 714（439 with BRAF wild-type） | Nivolumab + Relatlimab 63 (20–94)  Nivolumab 62 (21–90) | 416 / 298 | As noted in the prior report,10 eligible patients were  12 years of age and had previously untreated, histologi- cally confirmed, unresectable stage III or IV melanoma and an Eastern Cooperative Oncology Group performance status of 0 or 1. Full eligibility criteria are provided in the previous report and can be found in Sections 6.1 and 6.2 of the study protocol, available with the full text of this article at evidence.nejm.org. | M0，M1 and normal LDH level Nivolumab + Relatlimab 104(232) Nivolumab 130(237) M1 and elevated LDH level Nivolumab + Relatlimab 76(123) Relatlimab 81(122) | 0 Nivolumab + Relatlimab 108（236） Nivolumab 136（242） 1 Nivolumab + Relatlimab 72（119） Nivolumab 75（117） | ≤ULN Nivolumab + Relatlimab 100（224） Nivolumab 127（231） >ULN Nivolumab + Relatlimab 79（130） Nivolumab 84（128） ≤2 × ULN Nivolumab + Relatlimab 158（322） Nivolumab 186（328） >2 × ULN Nivolumab + Relatlimab 21（32） Nivolumab 25（31） | NA | ≥1% Nivolumab + Relatlimab 68 (146) Nivolumab 67 (147) ＜1% Nivolumab + Relatlimab 112（209） Nivolumab 144（212） ≥5% Nivolumab + Relatlimab 33（88） Nivolumab 36（86） ＜5% Nivolumab + Relatlimab 147（267） Nivolumab 175（273） | nivolumab and relatlimab | 219 | Nivolumab 480 mg and Relatlimab 160 mg | Intravenous injection | Every 4 weeks | 42 month | nivolumab | 220 | Nivolumab 480 mg | Intravenous injection | every 4 weeks | 42 month | os： nivolumab and relatlimab vs nivolumab  Unstratified Hazard Ratio  os：0.83 (0.63 to 1.09)  pfs：0.78 (0.61 to 0.99)  orr：9.7 (0.6 to 18.6)  Nivolumab+Relatlimab (n=355)  Adverse events 352，Grade 3/4：154，Nivolumab (n=359)  Adverse events 344，Grade 3/4：126 | RELATIVITY-­047 NCT03470922). |
| Arance | 2025 | Eligible | 119 sites globally | 674（426 with BRAF wild-type） | Lenvatinib + pembrolizumab63 (19-89)  Placebo + pembrolizumab63 (24-87) | 410 / 264 | .Eligible partici­ pants were aged ≥18 years, had unresectable stage III or IV melanoma per American Joint Committee on Cancer eighth edition guidelines,10 had measurable disease per RECIST v1.1 by central review,11 and an Eastern Cooperative Oncology Group (ECOG) performance status of 0 or 1. Participants could not have received prior treatment for advanced or metastatic melanoma except for first-line BRAF inhibitors with or without MEK inhibitor therapy in participants with BRAF V600 mutation-positive disease. Participants with mucosal melanoma were eligible but were limited to ≤20% of the population; participants with ocular melanoma were excluded. | M0 Lenvatinib+ pembrolizuma 21 (6.3) pembrolizuma 18 (5.3) M1 Lenvatinib+ pembrolizuma 5 (1.5) pembrolizuma 1 (0.3) M1a Lenvatinib+ pembrolizuma49 (14.7) pembrolizuma66 (19.4) M1b Lenvatinib+ pembrolizuma78 (23.4) pembrolizuma88 (25.9) M1c Lenvatinib+ pembrolizuma 151 (45.2) pembrolizuma 138 (40.6) M1d Lenvatinib+ pembrolizuma 30 (9.0) pembrolizuma 29 (8.5) | 0 Lenvatinib+ pembrolizuma 234 (70.1) pembrolizuma 235 (69.1) 1 Lenvatinib+ pembrolizuma 100 (29.9) pembrolizuma 105 (30.9) | ≤ULN Lenvatinib+ pembrolizuma 203 (60.8) pembrolizuma 217 (63.8) >ULN but <2× ULN Lenvatinib+ pembrolizuma 97 (29.0) pembrolizuma 95 (27.9)≥2× ULN Lenvatinib+ pembrolizuma 28 (8.4) pembrolizuma 25 (7.4) Missing Lenvatinib+ pembrolizuma 6 (1.8) pembrolizuma 3 (0.9) | NA | CPS ≥ 1% Lenvatinib+ pembrolizuma 158 (47.3) pembrolizuma 174 (51.2) CPS < 1%/unknown Lenvatinib+ pembrolizuma 146 (43.7) pembrolizuma 134 (39.4) Not reported Lenvatinib+ pembrolizuma 30 (9.0) pembrolizuma 32 (9.4) | lenvatinib plus pembrolizumab | 211 | Lenvatinib 20 mg and Pembrolizumab 200 mg | Lenvatinib administered orally Pembrolizumab administered intravenously | Lenvatinib once daily; Pembrolizumab every three weeks | 46 month | placebo plus pembrolizumab | 215 | Placebo 20 mg Pembrolizumab 200 mg | Placebo orally Pembrolizumab intravenously | Placebo once daily Pembrolizumab every 3 weeks | 46 month | os： lenvatinib plus pembrolizumab vs placebo plus pembrolizumab  Hazard ratio (95% CI) 1.31 (1.01-1.71)  pfs：Hazard ratio (95% CI)  0.87 (0.68-1.13)  Lenvatinib plus Pembrolizumab (n = 332): Adverse events reported in 322 cases, with 195 grade 3–5 events. Placebo plus Pembrolizumab (n = 338): Adverse events reported in 293 cases, with 98 grade 3–5 events. | LEAP-003(NCT03820986) |
| Jedd D. Wolchok | 2025 | Eligible | 137 centers globally | 945（647 with BRAF wild-type） | Nivolumab alone 58.7 （25–90）#  Nivolumab plus Ipilimumab 59.3 （18–88）#  Ipilimumab alone 60.8 （18–89）# | 610 / 335 | Eligible patients were aged 18 years; had previously untreated, histologically confirmed, unresectable, advanced, stage III or stage IV melanoma; known BRAF V600 mutation status; and an Eastern Cooperative Oncology Group performance status of 0 or 1 (a 5-point scale where higher numbers reflect greater disability). Full study design and eligibility criteria have been described previously.6, 9,11–13 | M1c Nivolumab 184 (58.2) Nivolumab plus Ipilimumab 181 (57.6) Ipilimumab 183 (58.1) M0, M1a, or M1b Nivolumab 132 (41.8) Nivolumab plus Ipilimumab 133 (42.4) Ipilimumab 132 (41.9) | 0 Nivolumab 238 (75.3) Nivolumab plus Ipilimumab 230 (73.2) Ipilimumab 224 (71.1) 1 Nivolumab 77 (24.4) Nivolumab plus Ipilimumab 83 (26.4) Ipilimumab 91 (28.9) 2 Nivolumab 1 (0.3) Nivolumab plus Ipilimumab 0 Ipilimumab 0 Not reported Nivolumab 0 Nivolumab plus Ipilimumab 1 (0.3) Ipilimumab 0 | ULN Nivolumab 196 (62.0) Nivolumab plus Ipilimumab 199 (63.4) Ipilimumab 194 (61.6) >ULN Nivolumab 112 (35.4) Nivolumab plus Ipilimumab 114 (36.3) Ipilimumab 115 (36.5) 2× ULN Nivolumab 271 (85.8) Nivolumab plus Ipilimumab 276 (87.9) Ipilimumab 279 (88.6) >2× ULN Nivolumab 37 (11.7) Nivolumab plus Ipilimumab 37 (11.8) Ipilimumab 30 (9.5) Unknown Nivolumab 8 (2.5) Nivolumab plus Ipilimumab 1 (0.3) Ipilimumab 6 (1.9) | Yes Nivolumab 8 (2.5) Nivolumab plus Ipilimumab 11 (3.5) Ipilimumab 15 (4.8) No Nivolumab 308 (37.5) Nivolumab plus Ipilimumab 303 (96.5) Ipilimumab 300 (95.2) | Positive Nivolumab 80 (27.8) Nivolumab plus Ipilimumab 68 (24.5) Ipilimumab 75 (27.1) Negative Nivolumab 208 (72.2) Nivolumab plus Ipilimumab 210 (75.5) Ipilimumab 202 (72.9) | nivolumab plus ipilimumab | 213 | Nivolumab 1 mg/kg plus Ipilimumab 3 mg/kg, followed by Nivolumab 3 mg/kg | Intravenous injection | Nivolumab plus ipilimumab every 3 weeks for 4 doses (induction phase); Nivolumab every 2 weeks (maintenance phase) | 120 month | Nivolumab (with ipilimumab-matching placebo) Ipilimumab (with nivolumab-matching placebo) | 216      218 | Nivolumab 3 mg/kg with Ipilimumab-matching placebo; Ipilimumab 3 mg/kg with Nivolumab-matching placebo | Intravenous injection | Nivolumab administered every two weeks (plus Ipilimumab-matched placebo) Ipilimumab administered every three weeks for four doses (plus Nivolumab-matched placebo) | 120 month | os：NIVO+IPI  vs IPI 0.58(0.46-0.73)  NIVO+IPI vs NIVO  0.95(0.74-1.21)  NIVO vs IPI 0.61(0.49-0.77)   Melanoma-Specific Survival:  NIVO+IPI  vs IPI 0.51(0.40-0.66)  NIVO+IPI vs NIVO 0.88(0.67-1.15)  NIVO vs IPI 0.58(0.46-0.75) | The CheckMate 067(NCT01844505) |

*indicates values in parentheses represent the median and interquartile range.

#indicates values in parentheses represent the mean and interquartile range.

**Supplementary Table 3** League table and SUCRA ranking for OS

| Ipilimumab(SUCRA=22.37%,RANK8) | 0.7 (0.52, 0.94) | 1.33 (0.95, 1.86) | 0.61 (0.49, 0.77) | 0.9 (0.57, 1.44) | 0.9 (0.73, 1.11) | 0.51 (0.35, 0.72) | 0.92 (0.62, 1.36) | 0.58 (0.46, 0.73) |
| --- | --- | --- | --- | --- | --- | --- | --- | --- |
|  | Pembrolizumab(SUCRA=66.03%,RANK4) | 1.9 (1.21, 2.97) | 0.87 (0.6, 1.26) | 1.29 (0.9, 1.85) | 1.29 (0.89, 1.85) | 0.72 (0.45, 1.15) | 1.31 (1, 1.7) | 0.83 (0.57, 1.21) |
|  |  | Dacarbazine(SUCRA=3.14%,RANK9) | 0.46 (0.36, 0.59) | 0.68 (0.38, 1.21) | 0.68 (0.46, 1.01) | 0.38 (0.26, 0.55) | 0.69 (0.41, 1.16) | 0.44 (0.29, 0.66) |
|  |  |  | Nivolumab (SUCRA=76.52%,RANK3) | 1.48 (0.88, 2.49) | 1.48 (1.08, 2.01) | 0.83 (0.63, 1.09) | 1.5 (0.95, 2.38) | 0.95 (0.69, 1.32) |
|  |  |  |  | Epacadostat and Pembrolizumab(SUCRA=35.24%,RANK6) | 1 (0.6, 1.66) | 0.56 (0.31, 1) | 1.02 (0.65, 1.58) | 0.64 (0.38, 1.08) |
|  |  |  |  |  | Ipilimumab 10 mg/kg(SUCRA=36.77%,RANK5) | 0.56 (0.37, 0.85) | 1.02 (0.65, 1.59) | 0.64 (0.47, 0.88) |
|  |  |  |  |  |  | Nivolumab and Relatlimab(SUCRA=93.94%,RANK1) | 1.81 (1.06, 3.08) | 1.15 (0.75, 1.75) |
|  |  |  |  |  |  |  | Lenvatinib and Pembrolizumab(SUCRA=32.93%,RANK7) | 0.63 (0.4, 1) |
|  |  |  |  |  |  |  |  | Nivolumab and Ipilimumab(SUCRA=83.06%,RANK2) |

**Supplementary Table 4** League table and SUCRA ranking for OS after exclusion of Wolchok 2025 (1/2)

| Ipilimumab(SUCRA=20.78%,RANK5) | 0.7 (0.52, 0.94) | 0.9 (0.57, 1.43) | 0.9 (0.73, 1.11) | 0.91 (0.62, 1.36) |
| --- | --- | --- | --- | --- |
|  | Pembrolizumab(SUCRA=95.02%,RANK1) | 1.29 (0.9, 1.84) | 1.29 (0.9, 1.85) | 1.31 (1.01, 1.71) |
|  |  | Epacadostat and Pembrolizumab(SUCRA=44.31%,RANK3) | 1 (0.6, 1.66) | 1.01 (0.65, 1.58) |
|  |  |  | Ipilimumab 10 mg/kg(SUCRA=48.90%,RANK2) | 1.02 (0.65, 1.59) |
|  |  |  |  | Lenvatinib and Pembrolizumab(SUCRA=40.99%,RANK4) |

**Supplementary Table 5** League table and SUCRA ranking for OS after exclusion of Wolchok 2025 (2/2)

| Dacarbazine(SUCRA=0,RANK3) | 0.46 (0.36, 0.59) | 0.38 (0.26, 0.55) |
| --- | --- | --- |
|  | Nivolumab (SUCRA=54.54%,RANK2) | 0.83 (0.63, 1.09) |
|  |  | Nivolumab and Relatlimab(SUCRA=95.46%,RANK1) |

**Supplementary Table 6** League table and SUCRA ranking for PFS (1/3)

| Ipilimumab(SUCRA=5.79%,RANK2) | 0.4 (0.12, 1.38) |
| --- | --- |
|  | Nivolumab and Ipilimumab(SUCRA=94.21%,RANK1) |

**Supplementary Table** **7** League table and SUCRA ranking for PFS (2/3)

| Dacarbazine(SUCRA=0,RANK3) | 0.42 (0.33, 0.53) | 0.33 (0.23, 0.46) |
| --- | --- | --- |
|  | Nivolumab (SUCRA=51.14%,RANK2) | 0.78 (0.61, 0.99) |
|  |  | Nivolumab and Relatlimab(SUCRA=98.86%,RANK1) |

**Supplementary Table 8** League table and SUCRA ranking for PFS (3/3)

| Pembrolizumab (SUCRA=35.08%,RANK2) | 1.02 (0.79, 1.31) | 0.87 (0.67, 1.12) |
| --- | --- | --- |
|  | Epacadostat and Pembrolizumab(SUCRA=31.55%,RANK3) | 0.85 (0.6, 1.22) |
|  |  | Lenvatinib and Pembrolizumab(SUCRA=83.38%,RANK1) |

**Supplementary Figure 1** Convergence diagnostic plot for OS.

**Supplementary Figure 2** Convergence diagnostic plot for OS after exclusion of Wolchok 2025.

**Supplementary Figure 3** Convergence diagnostic plot for OS after exclusion of Wolchok 2025.

**Supplementary Figure 4** Convergence diagnostic plot for PFS (a).

**Supplementary Figure 5** Convergence diagnostic plot for PFS (b).

**Supplementary Figure 6** Convergence diagnostic plot for PFS (c).

**Supplementary Figure 7** Network plot for ORR (a).

**Supplementary Figure 8** Network plot for ORR (b).
